# The bright side of social network sites: On the potential of online social capital for mental health

**DOI:** 10.1177/20552076221093133

**Published:** 2022-04-12

**Authors:** Felix S Hussenoeder

**Affiliations:** Institute of Social Medicine, Occupational Health and Public Health, 9180 Leipzig University, Leipzig, Germany

**Keywords:** Social network sites, online social capital, theory, mental health, digital resources

## Abstract

Social network sites are an essential part of the daily lives of people around the globe, but our theoretical understanding of the phenomenon is still limited. However, to fully grasp the potential of social network sites and to be able to generate meaningful applications, a theoretical understanding of the phenomenon is needed. I want to introduce the concept of online social capital as the first step in this direction, and show how it could be applied to the area of mental health. Therefore, I will (1) bring together social network sites and social capital theory, (2) introduce online social capital with a special focus on its capacity and mobilization as well as on associated processes of relationship maintenance and information search, (3) explore potential implications for mental health promotion, (4) depict the mental health risks that are associated with the use of social network sites, and (5) highlight some areas for future research.

Social network sites (SNSs) have become an essential part of the daily lives of people around the globe. While there is no shortage of empirical research, our theoretical understanding of the technology is still limited. In fact, most of the time, theoretical approaches and concepts from traditional “offline research” are merely applied to the digital realm without any form of adaption. Hence, SNSs are treated like a digitalized form of something already known, similar to other media, offline relationships, or social situations. However, to fully understand the potential of SNSs and to be able to generate new and innovative research ideas as well as meaningful real-world applications, a theoretical understanding is needed that takes into account the unique and digital nature of the phenomenon. Therefore, I want to introduce the concept of online social capital (OSC) as the first step in this direction. I will therefore give a short overview on those elements of the social capital tradition that are helpful for understanding the digital space, discuss the differences between traditional networks and those on SNSs, and introduce the concept of OSC as well as the processes associated with it. I will further show how OSC can be used to promote mental health and well-being, depict the mental health risks that are associated with OSC, outline some potential avenues for future research, and end with a conclusion.

## SNSs and social capital

SNSs can be seen as “bundles of technological tools that incorporate features of earlier technologies (such as personal websites) but recombine them into a new context that supports users’ ability to form and maintain a wide network of social connections”.^1^ Besides their basic commonalities, SNSs exhibit many differences with regard to their popularity, the more or less specific group of people they address (e.g. business people, photo enthusiasts, researchers, etc.), functions (career, dating, knowledge sharing, etc.), costs, designs, and specific features. The emphasis of this article is on rather unspecific SNSs where users connect with a wide array of diverse contacts, from family and close friends to acquaintances, to pursue a diverse set of (online) activities like commenting, sharing pictures, or engaging in discussions. At this time, Facebook is the prototypical example for that kind of multipurpose SNS, but there are others like WeChat or VK.

While SNSs combine multiple functions, their most prominent feature is the network. The personal, interactive, and digital networks associated with SNSs are not only a defining element, they are also crucial for understanding the potential and implications of SNSs. With its focus on networks and resources, the social capital metaphor is a useful concept for understanding the phenomenon. The concept is flexible enough to match the theoretical and conceptual needs of a new technology that is permanently changing, and to help bring together the perspectives of multiple disciplines.

### Social capital

Although the term “social capital” has been used since 1916,^2^ its popularity among scholars started in the 1980s with French sociologist Pierre Bourdieu^3^ who used the concept to explain the persistence of social inequalities over time. To date, the concept has been applied to many different fields and disciplines from political sciences^4^ to economics,^5^ and over the time, it has been used to describe a variety of different phenomena and to explain social trust as well as personal success. The focus of this article is on a particular strand of theorizing about social capital known as the “network-based utilitarian school”^6^ since that school addresses individual networks, resources, and actions. A more comprehensive discussion of the social capital concept, the different traditions, including the critical and the normative school of thought, and their application to social media can be found elsewhere.^7^

Based on his extensive review of the literature, Lin^8^ defines social capital as the “resources embedded in a social structure which are accessed and/or mobilized in purposive actions” (p. 35). In his view, resources comprise wealth, power, or status and they are directly or indirectly accessible via one's social connections. Lin^9^ can be credited for developing a structured approach that clearly differentiates between three main segments of social capital that are often confounded in the literature: (1) an actor’s position in a network, (2) the mobilization of resources, and (3) its effects. In addition, he distinguished between the potential access to social capital, i.e. capacity, and its mobilization. While capacity refers to the size and diversity of resources embedded in digital networks, mobilization includes processes like information search and communication. Both are facilitated, intensified, and shaped by digital technology and closely linked to mental health outcomes. The differentiation between the two as well as Lin`s broad, individual-level approach to social capital will become central to the approach presented in this article.

The network-based utilitarian school puts its focus on the qualities of social structures, and compared to previous scholars like R. Putnam and J. S. Coleman,^4,10^ shifts attention away from the societal/political level to an individual one. This makes the approach especially valuable from a psychological perspective. In addition, scholars in this tradition put an emphasis on the link between structure and access to information. They argue that actors that connect different groups may benefit from that position as they have access to nonredundant sources of information among other benefits,^11,12^ an argument that is reminiscent of the seminal work of M. S. Granovetter on the “strength of weak ties”.^13,14^

The network-based utilitarian school provides researchers with distinct definitions that are oriented toward empirical validation, which makes it a useful approach for application. Two major points of criticism is that the school ignores those aspects of social capital that are difficult to operationalize and assess, like trust or implicit norms, and the potential negative effects of social capital.^6,15^ Compared to the critical and the normative school of thought, the network-based utilitarian school applies an individualistic focus. With its emphasis on measurable variables and clear definitions, and its appreciation for network structure, it is no surprise that the network-based utilitarian school left a strong imprint on social media research.

### Social capital on SNSs

The social capital concept has been applied by multiple SNS scholars over the time (e.g.^16–18^) and most of the literature distinguishes between two forms: (1) bonding social capital, which is related to close relationships like those from family and close friends and (2) bridging social capital, which is rooted in large and heterogeneous networks. The conceptual differentiation goes back to the work of the sociologist and political scientist R. Putnam,^19^ a highly influential researcher from the normative school. Bonding and bridging social capital were operationalized for SNS research and popularized by D. Williams.^20^ While bonding social capital is related to emotional support, bridging social capital is usually improving an actor's ability to access information resources. In addition, Ellison, Steinfield, and Lampe^17^ introduced *maintained social capital* which refers to connections from before college, e.g. those with high school acquaintances, which can be a source of information or small favors. However, this category seems unnecessary since its functional and structural differentiation from bridging social capital remains unclear, which is probably the reason why the concept is no longer popular in the literature.

The vast majority of research in the area is focused on the empirical connection between some form of social media use and the generation, existence, or maintenance of social capital. In their meta-analysis, Liu et al.^21^ show a positive connection between SNS use and both forms of social capital, and Williams^22^ in his current review confirms the connection between SNS use and bonding social capital.

A quick search on platforms like PubMed, Web of Science, or Google Scholar reveals a huge amount of literature on SNSs under the label of social capital. Despite a large number of studies from different disciplines like Psychology, Sociology, Political Sciences, or Economics that address a wide range of topics from social commerce,^23^ professional identity,^24^ and political participation^25^ to well-being^26^ and depression,^27^ a coherent theoretical approach that takes into account the specific nature of SNSs is still missing. That is, most of the time researchers treat social capital as an outcome and SNS use as one potential impact factor. Hence, they ignore the fact that because of the underlying technology digital networks are fundamentally different from traditional networks, e.g. with regard to size and inclusiveness, and that some forms of resources as well as the ways to access and utilize them only exist in the digital realm. However, to fully understand the nature of the phenomenon, an SNS-specific understanding of social capital is needed.

## OSC—an SNS-based resource

I define OSC as the resources that SNS users can directly or indirectly access via their SNS networks. For example, these resources can be in the form of expertise, opinion, or support. Research showed that network size and SNS use were positively associated with OSC, and that users with more OSC were more likely to utilize the SNS for coping-related information seeking as well as more satisfied with the SNS.^7^

From my perspective, it is the combination of four central properties that make SNSs different from all previous forms of social networking. Most obviously, they are (1) technology-based, which makes them scalable, permanently available, and easy to manage, browse, and access. It also means that they are to a large extent independent of time and place, as long as there is a technological device and Internet connectivity. They are (2) transparent and information-rich, i.e. contacts and corresponding profile information are visible to a degree that is highly unlikely in the offline world.^28^ To some extent, this visibility also extents to contacts of contacts or even complete strangers. Depending on platform policies and user choice, profiles can include a wide range of information from a profile picture, age, gender, location, and a personal list of contacts to hobbies, sexual preferences, and political beliefs. They are (3) communicative, i.e. SNS users can communicate with anyone inside their network (and beyond) via a communicative infrastructure with little effort and without any additional contact details.^7^ This includes one-to-one communication as well as one-to-many. The infrastructure includes actors like companies, organizations, clubs, institutions, and celebrities. Moreover, communication is not only facilitated by the technology, but also actively encouraged, e.g. when a newsfeed provides users` with the latest content from their network or reminds them of someone’s birthday. SNSs are (4) inclusive, i.e. they contain contacts from many different areas and times of a user’s life, including ephemeral contacts that would have been lost without the technology.^29^ In addition, they are inclusive on a much more fundamental level as anyone can join SNSs, and there are few entry criteria like internet access or minimum age. The combination of the four properties mentioned above clearly differentiates SNSs from their offline ancestors, and it further demonstrates the need for an SNS-specific social capital definition.

Based on Lin,^9^ a differentiation is made between the capacity and mobilization of social capital. This differentiation is important since the mere existence of a resource does not implicate that a person will also use it in a specific situation. For example, in some cases, actors may just not be aware of its existence, or the psychological threshold to asking for support may be too high.

### Capacity

The ways in which digital technology facilitates the initiation and maintenance of social networks boost the size and diversity of embedded resources, i.e. the capacity of OSC.

The initiation of digital relationships is almost effortless and supported by the technology that may even suggest potential new contacts. Since SNS contacts usually do not come with many strings attached, networks can easily grow. Once relationships had been initiated, they are maintained in two main ways, passively and actively.

*Passive relationship maintenance* refers to the fact that—after the initial connection—no active contribution from an SNS user is required to maintain relationships, allowing users to easily stay connected with contacts that may be distant in terms of time, space, or lifestyle. This form of maintenance is highly specific to SNSs, and it contributes to the inclusiveness that I previously identified as one of the four defining features of digital networks. In addition, via different technological features, i.e. a news feed, and through small exchanges of information, SNS networks achieve a subliminal presence in the minds of SNS users that contributes to the maintenance of relationships and that has been referred to as “ambient awareness”.^30^ By reducing the felt distance between contacts, ambient awareness can reduce the threshold for requesting information.

*Active relationship maintenance* refers to online social interactions like the exchange of texts, pictures, and other digitalized information between SNS users and (groups of) their contacts that are facilitated by the communicative infrastructure of SNSs. It could be seen as a technology-based form of traditional social interactions, but with time SNS-specific forms of communication evolved. For example, the “like” button is a Facebook-specific feature that allows users to comment on content with minimal effort. By liking a specific statement, event, or picture, users engage in a social interaction. While this may seem like a superficial way of relationship maintenance, it is actually a form of interaction that suits large networks and that can contribute to ambient awareness.

Passive and active forms of relationship maintenance can alternate, and they allow SNS users to build and maintain large networks with little effort. The relevance of those “low costs” becomes especially obvious when compared to the effort (mainly in terms of time and motivation) that would be required to maintain relationships with the same number of contacts without the help of technology. The role of technology in building and maintaining social relations is especially important with regard to contacts that are not close friends and family. For many of these contacts, without the technology, the “potential benefits of staying in touch are overwhelmed by the costs of coordination, making it unlikely that the connection persists”^29^ (p. 138). In other words, it is very likely that a person will stay connected with their close emotional relationships, with or without the use of SNSs. However, the cognitive effort and time that individuals are willing to invest to keep up relationships with distant contacts are limited. Due to the low costs for relationship maintenance on SNSs, users can afford to stay connected with even the weakest ties.^29^ Compared to other contacts, these ties are more likely to be different from the user with regard to lifestyle, experience, culture, occupation, hobbies, age, socioeconomic status, etc. The inclusion of those contacts, and the fact that they do not drop out of sight, increases the diversity of the network.

The capacity of OSC is based on the quantity of network connections as well as on their quality, e.g. in terms of diversity, expertise, or trustworthiness. Due to the technology-facilitated processes of relationship initiation and maintenance, the capacity of OSC is significantly bigger compared to non-digital networks and the capacity of traditional forms of social capital.

### Mobilization

To mobilize their resources and to receive information, like support or opinion, users have to know where in their network they can find someone with the specific knowledge and they have to as for that information, i.e. initiate communication. Therefore, two main technology-based strategies are relevant.

1. Profile information of contacts can be used to directly search for a contact that can provide a specific information. For example, a contact that lives in a certain area and indicates that on their profile could have information on flats or jobs in that area. Once this “expert” has been identified, they can easily be addressed via the communicative infrastructure of SNSs without any further contact data. In other words, SNS technology is lowering the transaction costs for social interactions.^29,31^
2. Requests can be communicated to a large group of contacts at the same time, e.g. via profile posts. This is especially useful when it is unclear who (or if anyone) in the network has a certain information or when users are interested in more interactive processes of knowledge generation like discussions or the collection of diverse opinions. This indirect searching strategy is seen as easy, reliable, informal, and sufficiently fast and efficient, while simultaneously satisfying social needs.^32^ The fact that contacts can easily share requests with their own personal networks increases the potential benefits of this search strategy. Additionally, groups on SNSs that are centered on specific topics or common interests can further facilitate indirect searching and improve outcomes.

Due to the pervasiveness of the communication technology, the felt proximity of contacts that is promoted by technology-facilitated interactions, and the mere visibility of the other, SNSs lower the psychological threshold for the initiation of communication. In other words, while most people would not pick up the phone to call someone they had met 10 years ago to ask for information that this person may not even have (given they even have the contact details), they are much more likely to send a request via SNS messenger or to post it on their profile. One could argue that there are other internet options, especially forums, that allow users to post requests and wait for replies, and that therefore SNSs are not a unique phenomenon, at least in the digital realm. While this is true to some extent, there are two significant differences between posting on an SNS and on a forum, which have a direct effect on the nature of information-seeking processes. First, SNSs are not topic specific (at least the ones that are at the focus of this article), allowing a wider set of requests without constantly changing platforms. Second, since SNS networks refer to personal contacts, trustworthiness and personal relatedness of replies are in general higher.

The described strategies make it easy for SNS users to find, ask for, and receive information or support, making the mobilization of OSC faster and more efficient than it would be in the case of offline social capital. These strategies benefit from the properties of digital networks, i.e. from the transparency and information richness of user profiles and the pervasive communicative infrastructure. Especially undirected search may strongly affect the efficiency and range of OSC mobilization. By posting a request on their profile, users are likely to receive replies from contacts who they may not have thought of before and thereby gain access to resources that would otherwise have remained unused. The low effort involved in posting a request and the permanent availability of OSC make this form of information seeking a useful option, even in cases when users do not know if anyone in their network has access to the requested knowledge. Therefore, many requests that users would not have asked for without the undirected search option, can now be posted. In other words, the mobilization of OSC is extended to areas and topics that go beyond those that are traditionally addressed by other forms of social capital, thereby extending the usefulness and applicability of users’ resources.

As I argued in the previous paragraphs, the technology provided by SNSs boosts the capacity as well as the mobilization of OSC compared to offline forms of social capital. Technology does not only increase the capacity of resources, but also their mobilization and applicability. The described processes of relationship maintenance, searching, and communication make digital networks an ideal tool for information seeking in the widest sense, and they give users access to an unprecedented amount of expertise, knowledge, and opinion.

## Potential mental health applications of OSC

OSC is a way of conceptualizing SNS-based resources that can be applied to better understand and improve the mental health of SNS users. It can be used to complement a research tradition on the negative associations between social media use and mental health (e.g.^33,34^) and to contextualize, integrate, and extend the literature on mixed and beneficial mental health aspects, outcomes, and interventions in the general population (e.g.^35,36^) as well as in people with mental health conditions like psychosis and depression^37–39^.

In addition, it can help researchers and practitioners to develop, implement, and evaluate mental health interventions and support in a systematic way. The concept can also be used to communicate complex mental health issues to lay audiences, politicians, and decision makers, and it can lay the foundation for further research and theory development.

In the following paragraphs, some ideas for the application of OSC will be presented. They are either targeted at increasing the capacity of OSC or at improving its mobilization. They are not meant as a conclusive list of projects, but rather represent a collection of ideas that show the potential of the concept to generate meaningful real-world applications. While some of the following suggestions could also be addressed with other forms of digital support like support groups, virtual reality chat, bulletin boards, or forums (e.g.^40^), SNSs have a crucial advantage. They are already an essential part of many people’s lives, it is where they spend time, inform themselves, and interact. Therefore, promoting mental health via OSC avoids the stigma associated with specific mental health offers, and it significantly lowers the threshold for participation. Moreover, mental health promotion is embedded in an enjoyable environment that can help to boost and maintain motivation, and health education and interventions can become a part of regular social media activities. Mental health providers can to some extent utilize the already existing technology, and the possibilities for outreach, dissemination, feedback, communication, and evaluation. As I will discuss later on, the ideas presented in this section require SNSs that are controlled by their users or at least guarantee a certain level of privacy and data protection to be meaningful, beneficial, trustworthy, and safe.

### Building OSC capacity

Building OSC capacity could be used to address stigmatization and lack of mental health knowledge which still create unnecessary barriers that prevent people from seeking help for mental health problems.^41^ However, seeking help at an early time is important to prevent more severe forms of mental ill-health, which benefits those affected as well as society as a whole via the reduction of treatment costs.

To build the capacity of OSC, one could increase the resources associated with contacts in the network. For example, this could be done by training specific SNS users to become “mental health influencers” in those SNS networks that they are a part of. They could regularly disseminate information on mental health topics and thereby normalize the topic, provide access to and information on support, and answer questions from contacts. Influencers could receive a specific training and have a mental health background and/or lived experience. The fact that they are part of the same network makes it easy for help seekers to address influencers directly, trust each other, and communicate. It also makes it more likely that users pay attention to the content they share and engage in meaningful communication. Technology could be used to increase the visibility of influencers, support the dissemination of their content, target specific audiences, and evaluate and improve their impact. Users could apply to become influencers or be nominated by their peers. There is also a potential to create SNS-based games that support mental health knowledge, like quizzes, and get users to engage with influencers.

Another way to build capacity could be the integration of new contacts in existing networks. For example, mental health professionals, psychotherapists, and social workers could be recruited to develop an SNS presence and connect with users. Similar to the influencers discussed above, they could distribute content and provide support. In addition, they could offer online and offline counseling, coaching, or therapy. This could significantly lower the threshold for seeking professional help.

### Mobilizing OSC

To improve OSC mobilization, an SNS application could be used to visualize certain areas of knowledge, expertise, or experience that exist in the network of a user. For example, some contacts may have information on “writing exams”, “dealing with stress”, or on “how to start your own business”. By visualizing these areas of knowledge and the corresponding experts, SNS users could easily ask for support. In that way, individuals can use SNSs to better cope with hassles in their daily lives, and thereby increase their well-being and mental health. Since their networks consist of personal contacts that to some extent know each other, the information they receive will be much more likely to match their needs and personal situation, i.e. more helpful than other online sources of information.

Another way to mobilize OSC would be through an application that allows SNS users to post questions with specific tag words which are then directed toward contacts that are able and willing to give information and/ or support. In that way, users can receive support from those contacts that are willing and able to help them. This form of information seeking could reduce the number of unwanted requests that contribute to the experience of social overload, and it could prevent the requester from rejection and low-quality information.

In addition, resources could be mobilized in an anonymous way, e.g. via an application that hides the identity of users while it allows them to ask others in their network and beyond for support or advice. This would lower the threshold for help seeking, especially when there is a high risk of stigmatization and/ or the topic is highly sensitive.

Another way of mobilizing OSC is by deriving diagnostic information from SNS users. SNS networks are virtual environments where multiple forms of interaction take place, mostly in front of a large and diverse audience, i.e. a network of contacts. As a consequence, changes in users’ digital behaviors can easily be recognized by their peers online. For example, a user may suddenly start to communicate about negative feelings, or change typical communication patterns without an obvious cause. In these cases, digital networks could be used to identify and address potential mental health problems at an early stage. For example, an application could be implemented that mobilizes OSC by allowing contacts to voice their concerns about a specific user. After a certain number of contacts indicated their concern about a specific user, that user would receive information on mental health resources and/ or support. Hence, the application would act as a tool to mobilize an information resource, i.e. in that case concern. By reducing the threshold for voicing concern and by aggregating the input from multiple sources, the application goes beyond what would be feasible in the offline realm.

This form of network-based diagnostics could be a promising, social complement to the technology-based analysis of social media activity as a potential indicator for mental health problems like depression and suicidality^42–44^. It could especially be useful to differentiate mental health-related changes in the patterns of user behaviors from forms of false alarms and “behavioral noise”. For example, where network-based diagnostics detect a problematic pattern of social withdrawal, contacts may see a person that just entered a new romantic relationship. In addition, users may identify problematic pictures much better than algorithms, and the information gained from contacts could be used to improve network-based diagnostics and to assist psychologists and other mental health experts with their diagnosis. In the long run, this information could be used to predict the incidence of mental ill-health and to develop personalized health interventions.

## Risks associated with OSC

OSC has great potential to benefit the mental health of SNS users, but there is an obvious problem. At the moment, the most popular SNSs do not offer the level of data protection and privacy that is required for an issue as sensitive as mental health. The business model of those SNSs is built on the exploitation of user data, and there is a huge power imbalance between those owning SNSs and those using them, leaving users with little control over their digital lives. In addition, there is a risk that data can be hacked and/or abused by criminals, intelligence agencies, and others. In the given situation, why should we nevertheless conceptualize OSC as a mental health resource? Because there are multiple alternatives to the currently popular SNSs that promote data protection and privacy, shift control to users, and, for example, are financed via donations (e.g. Friendica or WT.social). While it is not clear now which of those SNSs will become relevant in the future, they nevertheless represent a growing need (and supply) for a different form of digital social networking with a great potential for OSC and mental health. By developing, evaluating and promoting concepts and ideas researchers can contribute to that development, and they can accompany and stimulate digital innovation. In the meantime, popular SNSs can still be used as resources to some extent, e.g. for creating mental health awareness and distributing resources.

While data protection and control over onès data are the two elephants in the room, other threats to mental health exist on SNSs that have to be taken into consideration. First, social overload from SNSs can be linked to reduced well-being^45^ and increased distress.^46^ In a way, social overload can be seen as the direct flip side of OSC. The fact that SNSs facilitate users’ access to vast amounts of information and provide the technology to request support now turns against users which are overwhelmed by information and the demands from their large number of contacts. Just like in the cases of OSC capacity and mobilization, the technology of SNSs facilitates social overload to a degree that is unprecedented in the offline world. Second, there are different forms of cyber harassment, including online forms of bullying and stalking, which are connected to mental health,^47^ psychosocial problems, substance use, depressive symptoms, suicidal ideation, and suicide attempts.^48^ Third, the excessive and addictive use of social media had been linked to a reduction in life satisfaction and well-being,^49,50^ increased psychological distress,^51^ and poor sleep quality.^52^ Fourth, there is a huge potential for deleterious social comparisons due to the vast amount of social information online, e.g. in profiles, pictures, and posts. These comparisons are connected to lowered well-being^53^ and depression.^54^ Fifth, there are other phenomena on SNSs that may affect users’ mental health in more indirect ways, for example, hate speech and “social media bubbles”.

While these risks and problems also exist outside the digital realm, the same technology that facilitates OSC can also intensify the downsides of social media. For example, in the case of bullying, large networks that facilitate OSC capacity can increase the potential number of bullies, and the same communication technology that fosters the mobilization of OSC can accelerate the spread of defamation and rumors. With the widespread adoption of social media, improving internet connectivity around the globe, and the use of social media via mobile phones it is very likely that also these associated risks become more widespread.

## Future research and application possibilities

The OSC concept can help researchers and practitioners to better address public mental health issues, but it can also stimulate meaningful research and application. In the following paragraphs, I will introduce five potential avenues for the future.

First, in this article, I tried to develop a basic concept to analyze unprecedented, digital resources. Future research may continue these efforts, and focus on the prerequisites and outcomes of OSC, for example, on the connection between offline friend networks, personality, or social status and digital resources, or on the effects of OSC on well-being, depression, and dementia. Research could also help to better understand the factors that contribute to the capacity of OSC like contact expertise. It could shed light on the processes that allow users to mobilize their resources or prevent them from doing so.

Second, since there are severe mental health risks involved in the use of social media, future research may help to better identify, understand, and mitigate those risks. This could include the identification of risk groups, the development of safer use guidelines, or the evaluation of new SNSs. Researchers could help users to be aware of and to better balance the chances and risks of social media use and promote media competency, which is also an important precondition for the responsible use of digital technology for mental health.

Third, there is a need for new measures that go beyond those that are traditionally used in psychology and the social sciences. For example, measures like average degree centrality and the number of isolated contacts in a network could be interpreted as structural representations of diversity that may be relevant for the assessment of mental health resources (see Jackson^55^ for more information on the measurement of network structure). They offer a complement rather than an alternative to subjective, psychological measures and are already used by some researchers.^56^ In addition, network measures could reveal resources that are unknown even to the users themselves.

Fourth, there is a great potential for the development of interventions to benefit mental health of users, and future research may contribute to the development, implementation, and evaluation of SNS applications and even better SNSs. This calls for interdisciplinary research efforts that involve not only social scientists and psychologists, but also programmers, technology experts, and data analysts.

Fifth, research and political action are needed to develop legal frameworks and legislation that shift control to the users of SNSs and protect their data and privacy to create a foundation on which sensitive topics like mental health can be addressed.

## Conclusion

I shortly introduced the concept of social capital and its application to SNSs before presenting my own concept of OSC as a way to do justice to the uniqueness of resources embedded in digital networks. I described how far processes of active and passive relationship maintenance contribute to the capacity of OSC, while direct and indirect forms of search can support OSC mobilization. My argument is that these processes contribute to the larger capacity and better mobilization of OSC compared to offline networks. I also introduced ideas for the application of the OSC concept to improve public mental health, discussed the risks associated with these resources, and depicted some potential avenues for future research.

Far from being a comprehensive framework, this article wants to contribute to a vocabulary and an understanding that helps us to identify the processes and outcomes that matter when talking about mental health in the digital age. It is also meant as a toolbox for the development of meaningful social media interventions to improve public mental health, and a call for better platforms.
